# Balcony genioplasty: a novel technique for better esthetic results in patients with deep mentolabial fold

**DOI:** 10.1186/s40902-019-0190-8

**Published:** 2019-02-11

**Authors:** Seied Omid Keyhan, Behzad Cheshmi, Hamid Reza Fallahi, Mohammad Ali Asayesh, Tirbod Fattahi

**Affiliations:** 1 Isfahan, Iran; 2 Boroujerd, Iran; 3 Jundishapur University of Medical Sciences, Ahvaz, Iran; 4 Dental Research Institute, Shahid Beheshti University of Medical Sciences, Tehran, Iran; 5 Division of Oral and Maxillofacial Surgery, University of Florida, Jacksonville, FL USA

**Keywords:** Esthetic, Surgery, Oral, Maxillofacial, Genioplasty, Short face, Mentolabial, Fold, Lip

## Abstract

**Background:**

To introduce a novel technique for advancement genioplasty helping surgeons to avoid soft tissue difficulties especially in short-faced patients with deep mentolabial fold and everted lower lip.

**Case presentation:**

In a trapezius-shaped, osteotomy was performed in the chin region. The mobilized segment was advanced, and the existing gap was grafted using interpositional allograft materials. Each side had been fixated by three-hole plates and two screws. The outcomes revealed no change in lower anterior teeth vitality. The patients did not report any changes of sensation in lower lip and chin either. The measurements indicated no increase in depth of mentolabial fold in patients undergoing this surgical technique. The postoperative evaluation showed a successful esthetic outcome for the patient and the surgeon concurrently.

**Conclusion:**

Based on our experience, the authors concluded that the Balcony technique is a simple and reliable procedure for patients with a deep mentolabial fold.

## Background

Multiple anatomic characteristics contribute to creating an esthetically pleasing and youthful appearing face, including skin, soft tissue, and facial bony contours [1]. The major architectural promontories of the facial skeleton, including the malar-midface region, nose, and chin, provide the base upon which the soft tissues of the face drape. By altering these promontories, dramatic changes can be made in the esthetic appearance of the face far more than by merely changing the soft tissue and skin alone [2]. The creation or restoration of an esthetically pleasing facial contour can encompass many surgical approaches. Several surgical techniques are available for correcting and giving harmony to the lower third of the face [3, 4].

In this respect, some well-known techniques seek to correct the shape and size of the chin using different kinds of chin implants or osteotomies in an effort to modify its spatial location, thus determining a new facial contour. Genioplasty is a versatile surgical technique which allows modifying the natural anatomy of the chin along all the three spatial directions. It was first described in the 1940s by Hofer, who referred to it as an “anterior horizontal osteotomy of the mandible.” In some patients in need of advancement genioplasty, a horizontal bony movement of the chin may result in unwanted changes in soft tissue envelope. This is especially true in short-faced patients with deep mentolabial fold and lower lip eversion; advancement genioplasty in this population may indeed accentuate the depth of the mentolabial fold [5–7].

The goal of this article includes introducing a novel technique for genioplasty (Balcony genioplasty) which can help surgeons avoid soft tissue esthetic difficulties especially in short-faced patients with deep mentolabial fold and everted lower lip.

## Methods

Informed consent was obtained for experimentation with human subjects. The patient’s face and mouth were prepared, and general anesthesia was administered, where local anesthesia was infiltrated by lidocaine 2% and epinephrine 1:100,000.

Genioplasty incision was made at 5 mm distance from the mucogingival line.

**Fig. 1:**
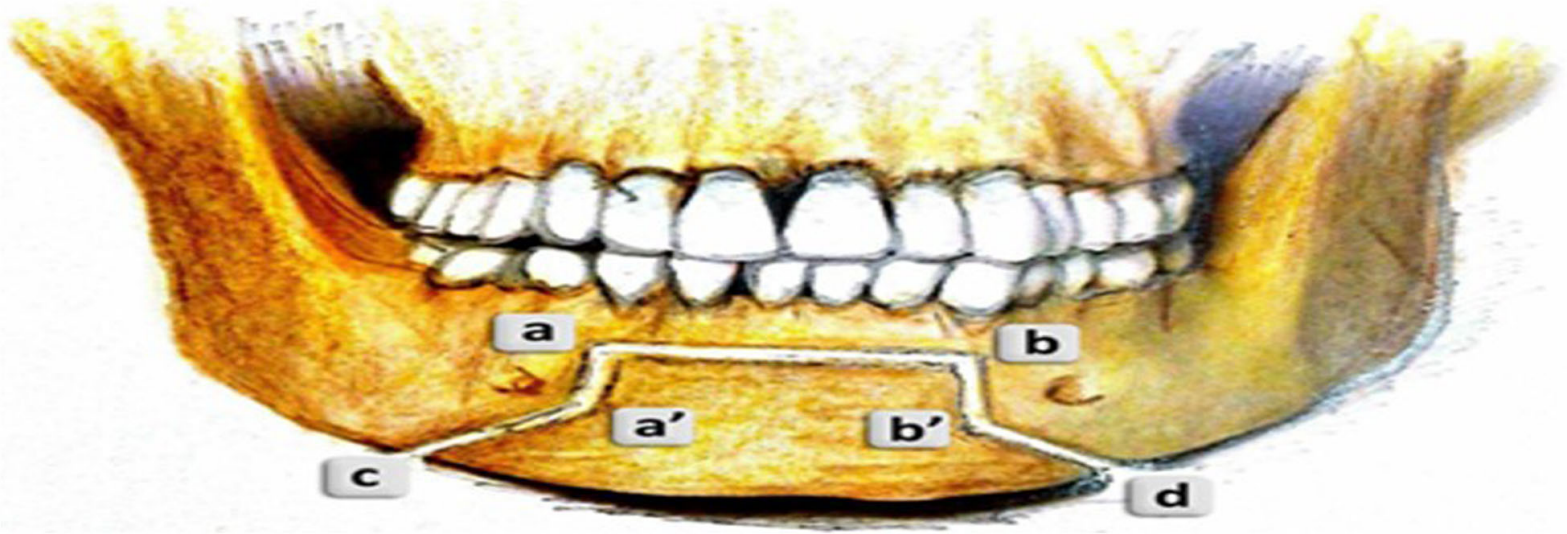
Schematic frontal view of cutting lines of Balcony technique

After bone marking, chin osteotomy with Balcony fashion was performed in two parts; superior rectangular part (from ab to a’b’ level) and a trapezius part (from a’b’ to cd level) (Fig. 1).

**Fig. 2:**
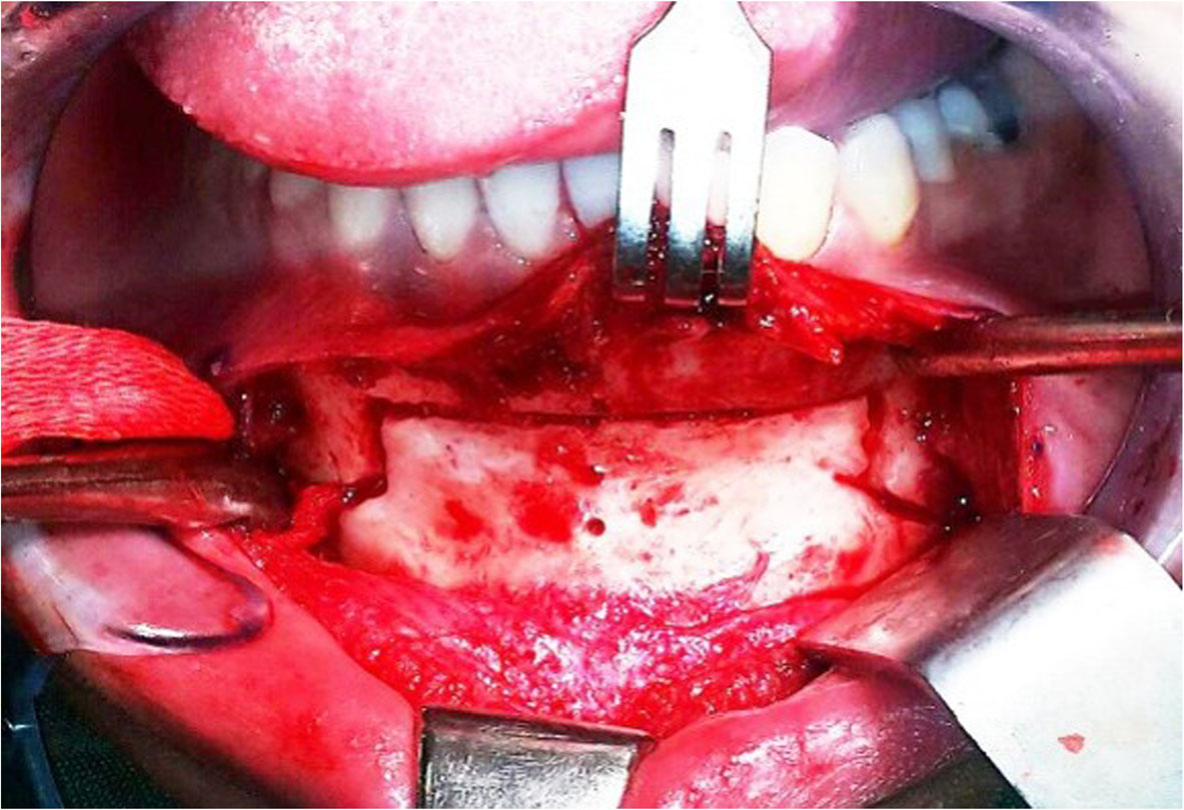
Intra-operative view of Balcony genioplasty

Osteotomy should be started in the superior part along (ab) line 5 mm away from canine apexes and be continued to the inferior line (a’b’). Only bone cortex and minimal cancellous bone should be separated from the rest of the bone and lingual cortex with the aid of the sagittal saw. It can be performed with angulation of 30–45° to the labial surface of lower incisors down to (a’b’) line with a swiping movement (Figs. 1 and 2).

Angles of the rectangular part were 90°. In the inferior trapezius part, the osteotomy was continued from (a’b’) to (cd) level, which is the inferior border of mandible. Osteotomy along lines (a’c) and (b’d) was continued similar to a routine genioplasty osteotomy while maintaining adequate space from mental nerve and mental foramen (Figs. 1 and 2).

**Fig. 3:**
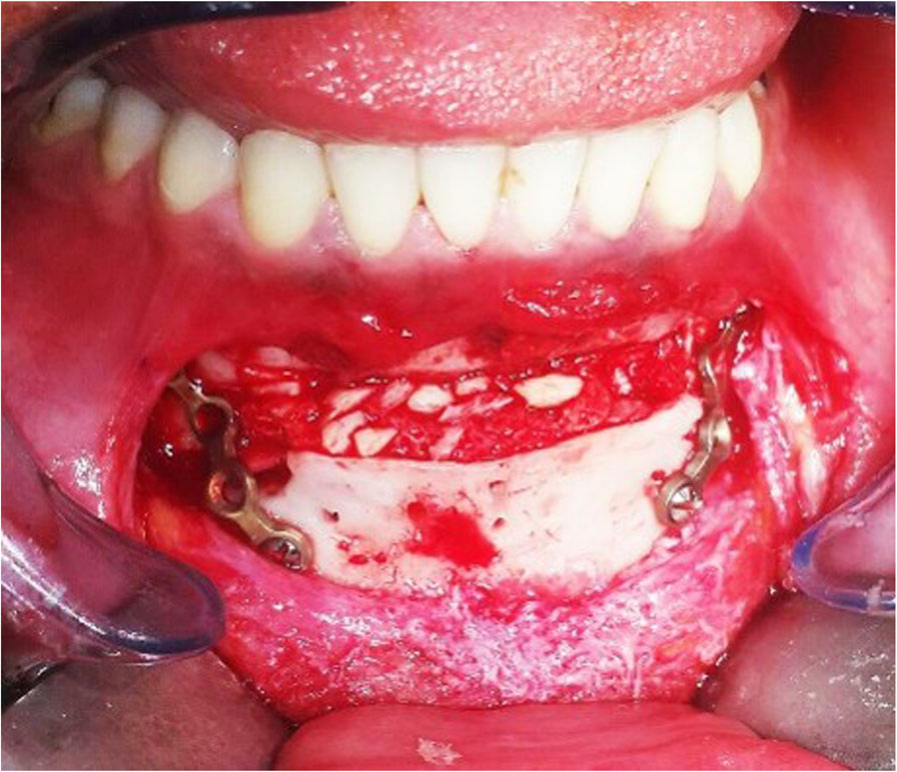
Intra-operative view of Balcony genioplasty

Note that the osteotomy at the level of (a’b’), the junction between upper and lower parts of the osteotomy design, should be done completely bi-cortically with maximum extension along (a’b’) level. Also, care must be taken to prevent unfavorable fractures of mono-cortical upper part. Meanwhile, c and d angles vary due to the level of augmentation required for the chin.

More acute angles lead to increased length of (a’c) and (b’d) lines and more extension beyond mental foramina, yielding a greater width along the inferior border, which is esthetically desirable in men [3]. On the other hand, if (c) and (d) angles are obtuse, it makes osteotomy lines (a’c) and (b’d) shorter, resulting in lower width in the chin and sharper view, which is esthetically desirable in women [3, 8].

The lengths of (bb’) and (aa’) are variable depending on the chin height, which is defined as the distance between the alveolar crest and Menton (Figs. 1, 2 and  3).

**Fig. 4:**
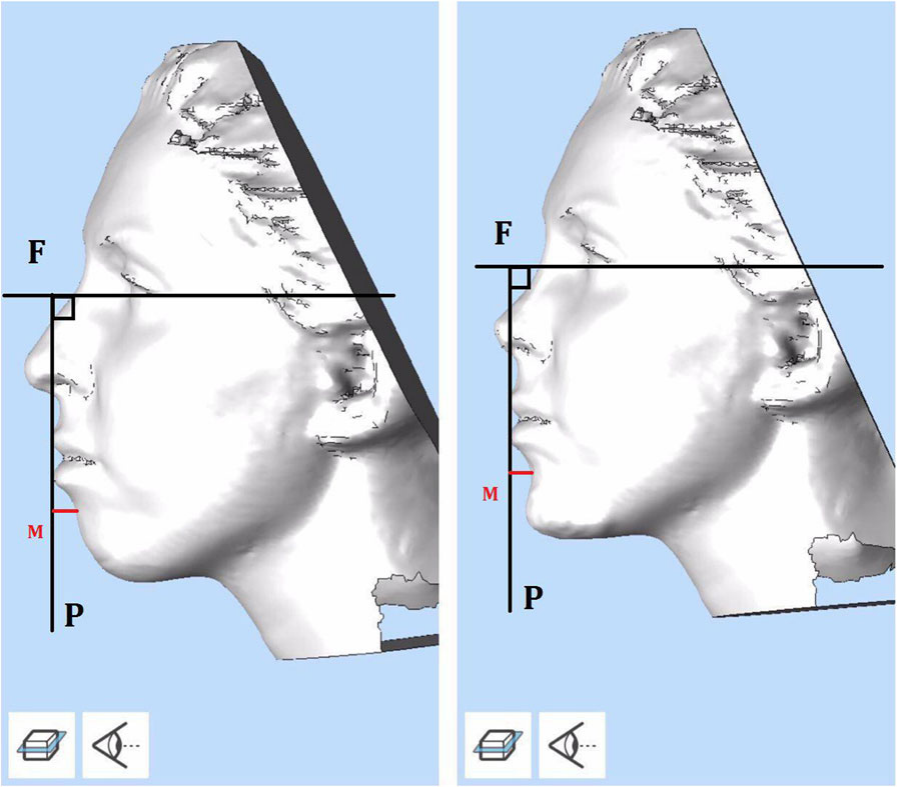
Pre (left) and post-surgical (right) 3D scan views; patient undergoing Balcony genioplasty and rhinoplasty; F, Frankfurt plane; P, perpendicular line to the Frankfurt plane; M, depth of the mentolabial fold

To determine the effect of this surgical technique on the depth of the mentolabial fold, the distance between the fold and the line perpendicular to the Frankfurt plane was measured in MeshLab® (http://meshlab.sourceforge.net, version 1.3.3), before and after surgery. For this purpose, facial 3D scans were also captured by Intel® RealSense™ before and after surgery (Fig. 4).

## Results

**Fig. 5:**
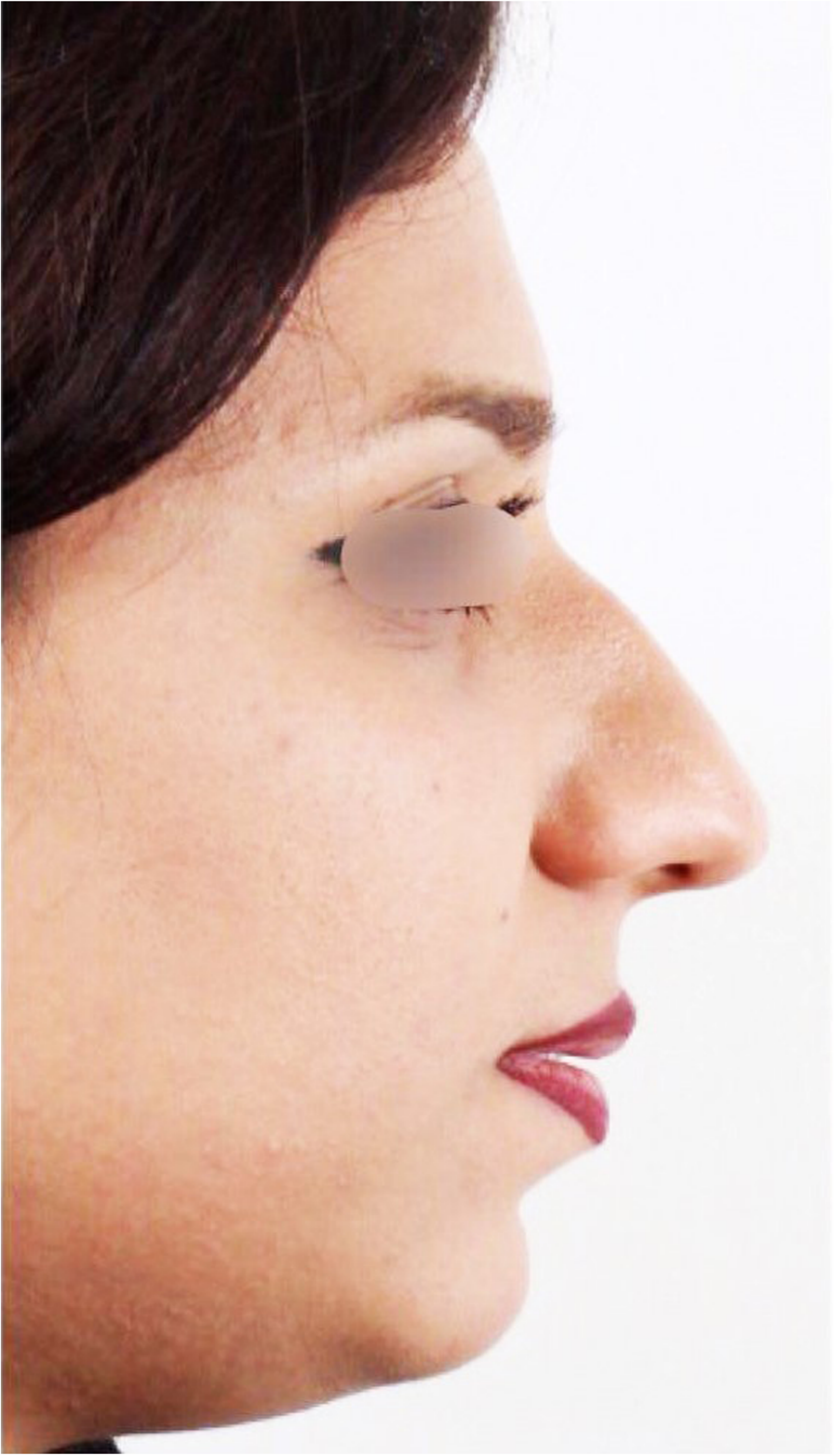
Preoperative profile view

**Fig. 6:**
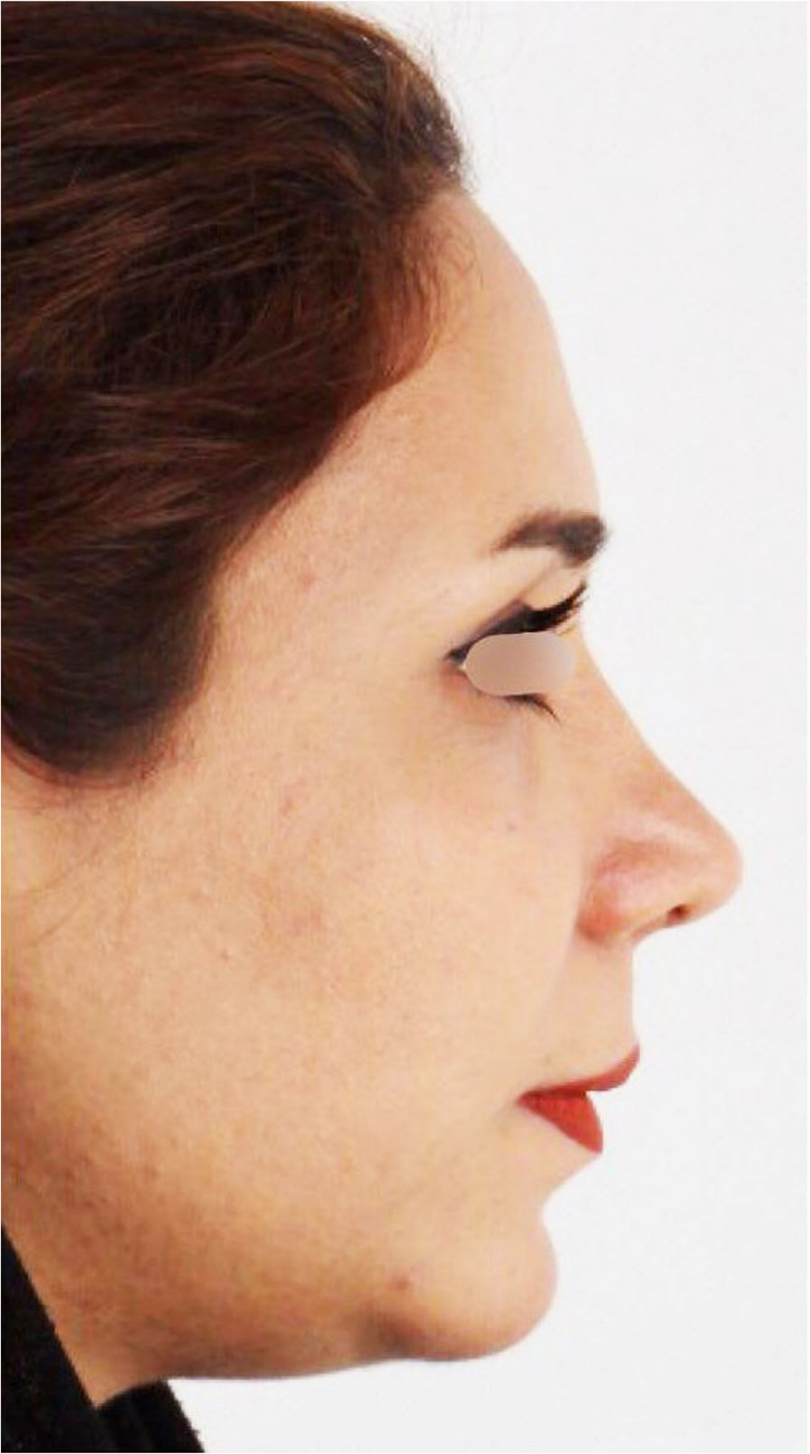
Postoperative profile view

The preoperative and postoperative views for one patient are shown in Figs. 5 and 6 respectively.

In total, 25 patients underwent the genioplasty procedure using our technique, out of whom, 21 were followed up for 1 year.

After the 1-year follow-up of our cases, esthetic outcomes of the technique were evaluated through radiographs and photos (Figs. 4, 5 and 6).

Clinical evaluations of patients revealed no sign of permanent neurosensory deficiency or muscular difficulties except in one case. Also, there were no symptoms of pain, and all patients were satisfied with the final result.

The vitality of lower incisors was tested by electronic pulp tester (Parkell, NY, USA). It revealed a positive response in mandibular incisors and canines as compared to maxillary incisors and canines as the control group. Based on our photographs, no elevation of the mentolabial depth was observed in any cases.

## Discussion

One of the advantages of Balcony techniques is avoidance of extra depth in mentolabial fold which makes unaesthetic appearance in routine techniques especially in short-faced patients with deep mentolabial fold and everted lower lip [8, 9].

Herein, we describe this technique using a sample patient. Note that other patients treated with Balcony technique and the same described protocol were esthetically satisfied. Further, the evaluation of the outcomes of each patient revealed no changes in sensitivity and teeth vitality and no side effects in soft tissues.

The ultimate goal of every cosmetic procedure is to maintain the function with enhanced esthetics. Based on the professional point view of the surgeon, the best indicator for a successful cosmetic procedure is patient satisfaction, where Balcony technique can accomplish this goal in multiple cases.

The use of Balcony technique due to parallel advancement of hard tissue in the inferior part of the mentolabial fold avoids extra deepening. Further, in cases with deep mentolabial fold, it results in augmentation and more filled look.

## Conclusions

Balcony technique is a simple and reliable procedure in patients with a deep mentolabial fold. This novel technique may help clinicians to expand their range of choices. This can lead to better esthetic results, which is the main goal of cosmetic procedures.
